# Perinatal excellence to reduce injury in preterm birth (PERIPrem) through quality improvement

**DOI:** 10.1136/bmjoq-2022-001904

**Published:** 2022-08-09

**Authors:** Alessandra Glover Williams, Sam Tuvey, Hayley McBain, Noshin Menzies, Sally Hedge, Sarah Bates, Karen Luyt, the PERIPrem Steering Group

**Affiliations:** 1 Neonatal Intensive Care Unit, University Hospitals Bristol and Weston NHS Foundation Trust, Bristol, UK; 2 Evaluation, South West Academic Health Science Network, Exeter, Devon, UK; 3 Project Management, West of England Academic Health Science Network, Bristol, UK; 4 Project Management, South West Academic Health Science Network, Exeter, UK; 5 Department of Paediatrics, Great Western Hospitals NHS Foundation Trust, Swindon, UK; 6 Bristol Medical School, University of Bristol School of Clinical Science, Bristol, UK

**Keywords:** Healthcare quality improvement, Obstetrics and gynecology, Paediatrics, Evidence-Based Practice, Infant Mortality

## Abstract

Perinatal Excellence to Reduce Injury in Premature Birth (PERIPrem) is an 11-element perinatal care bundle designed to improve outcomes for preterm babies, in line with the National Health Service (NHS) Long Term plan. Designed in collaboration with 12 NHS Trusts (secondary care hospitals), South West and West of England Academic Health Science Networks, South West Neonatal Operational Delivery Network, parent partners and clinical experts, implementation was via bespoke quality improvement (QI) methodology. Before project initiation, there was regional variation in uptake of elements, evidenced by baseline audit. Optimisation of the preterm infant is complex; eligibility for treatments is dependent on gestation and local policies. Preterm infants experience variability in care dependent on the place of birth, and there remains an implementation gap for several effective, evidence-based treatments.

The PERIPrem ambition is to reduce severe brain injury and death caused by prematurity by at least 50% through the delivery of a perinatal care bundle. The PERIPrem approach resulted in improved element implementation by 26% (from 3% to 29%) between 2019 and 2021, with dyads significantly more likely to receive the full bundle in 2021 compared with 2019 (probability=0.96 (95% CI 0.87 to 0.99), p<0.001). When examining the impact on psychological safety and team-working of PERIPrem, linear mixed models indicated an improvement in team function (p=0.021), situation monitoring (p=0.029) and communication within teams (p=0.002). Central to success was the development of a committed multiorganisational collaborative that continues to drive perinatal improvement using a common language and streamlining processes. In addition to saving the lives of the most vulnerable babies, PERIPrem aims to improve the chances of disability-free lives and is successfully nurturing high-functioning perinatal teams with enhanced QI skills.

**What is already known on this topic**

- The UK has one of the highest infant mortality rates in Western Europe; the 2019 National Health Service Long Term Plan aims to cut this and rates of neonatal severe brain injury by 50% by 2025.
- There is marked variation in execution of effective, evidence-based perinatal interventions across the UK.
- Quality improvement methodology has been found to be successful in its ability to implement and sustain perinatal practise change and reduce variation through programmes like PReCePT.

**What this study adds**

- Through bespoke quality improvement methodology and extensive organisational collaboration, a complex care bundle can be simultaneously, successfully and sustainably implemented over a large geographical area.
- Resultant of the quality improvement work, team-working, team function, situation monitoring and communication within teams all saw user-assessed improvement.

**How this study might affect research, practice or policy**

- A well-supported, remotely managed programme of quality improvement can result in widespread practise change with the employment of local champions.
- Quality improvement implementation of complex interventions is also associated with improvement in team function and communication.

## Problem

Preterm birth (before 37+0 weeks of pregnancy) is the single biggest cause of neonatal mortality and morbidity in the UK, constituting 24% of child deaths in 2019–2020.^1^ Despite a 13% reduction in neonatal mortality in England and Wales between 2010 and 2019, there remains significant variation in these figures across the UK.^2 3^

Although survival rates are improving for preterm babies, rates of severe disability have not followed the same trajectory, and there is now a growing population of children living with neurodisabilities, such as cerebral palsy, due to their prematurity. Research suggests that approximately 1 in 10 ex-preterm babies are classified as living with a severe impairment and 1 in 10 with a moderate impairment, with greater impairment as gestational age decreases.^4^

The National Health Service (NHS) Long Term Plan, itself responding to the Better Births publication, has committed to realising a 50% reduction in stillbirth, maternal mortality, neonatal mortality and serious brain injury by 2025, with an increased focus on preterm mortality.^5 6^ In order to achieve this, the Maternity and Neonatal Safety Improvement Programme (MatNeoSIP) included ‘improvement in the optimisation and stabilization of the very preterm infant’ as one of their five primary drivers. Perinatal Excellence to Reduce Injury in Premature Birth (PERIPrem) felt that it was crucial to maximise this opportunity to improve outcomes and implement an additional four evidence-based elements in addition to the seven already stated, focusing also on optimal timing of interventions to drive down neonatal mortality and brain injury. The resultant bundle is displayed further (table 1).

**Table 1:** Elements of the care bundle

Element	Description	Optimal timing	Evidence base
Place of birth	Babies <27^+^0 weeks’ gestation (<28^+^0 weeks’ multiples) or <800 g who are born in a tertiary neonatal intensive care unit (NICU)	n/a	Extremely preterm babies born in a non-tertiary unit are 2.3 times more likely to develop severe brain injury and 1.3 times more likely to die whether transported or not compared with controls.^24^
Antenatal steroids	Mothers who give birth at <34 weeks’ gestation receive at least one dose of antenatal steroids	Two doses 12–24 hours apart, >24 hours and <7 days prior to birth.	Reduces the risk of neonatal death by 31%, necrotising enterocolitis by 54% and grade 3–4 intraventricular haemorrhage by 46%.^25^
Magnesium sulfate	Mothers who give birth at <30 weeks’ gestation receive antenatal magnesium sulphate	>4 hours and <24 hours prior to birth	Reduces the risk of cerebral palsy by 32%.^26^
Intrapartum antibiotics	Mothers who are in active labour at any point prior to delivery receive intrapartum antibiotics	At least 4 hours prior to birth	Reduces risk of neonatal group B streptococcal sepsis in group B streptococcal colonised women by 86%.^27^ Reduces the risk of delivering within 48 hours by 29% and within a week by 21% and abnormal neonatal cranial ultrasound by 19%.^28^
Optimal cord management	Babies born at <34 weeks’ gestation have their cord clamped	At or after 1 min of birth	Reduces mortality by 32% compared with early cord clamping.^29^
Thermoregulation	Babies born at <34 weeks’ gestation have a normothermic temperature (36.5°C–37.5°C)	Within 1 hour of admission to the neonatal unit	28% increase in mortality per 1°C decrease in body temperature.^30^ Moderate hypothermia associated with higher odds of intraventricular haemorrhage (OR 1.3) and death (OR 1.5) compared with a normothermic temperature.^31^
Ventilation	Babies born at <34 weeks’ gestation who are in need of invasive ventilation are given volume-targeted ventilation in combination with synchronised ventilation as the primary mode of respiratory support.	On delivery	Reduces death or bronchopulmonary dysplasia by 27% and Intraventricular haemorrhage (grades 3–4) by 47% compared with pressure-limited ventilation modes.^32^
Caffeine	Babies born at <30 weeks gestation and/or <1500 g receive caffeine therapy	Within first 24 hours of life	The odds of death or clinical disability decrease by 40.2%.^33^
Early breast milk	Babies born at <34 weeks’ gestation receive first *maternal breast milk*	Within first 6 hours of life	Reduces the risk of necrotising enterocolitis by 38% compared with formula.^34^
Multistrain probiotics	Babies born at <32 weeks’ gestation and/or <1500 g are started on multistrain probiotic	Within first 24 hours of life	The odds of death are 44% less and the odds of developing necrotising enterocolitis are between 45% and 69% less when receiving probiotics compared with a placebo.^35^
Prophylactic hydrocortisone	Babies born at <28 weeks’ gestation are started on hydrocortisone	Within first 24 hours of life	The odds of survival without bronchopulmonary dysplasia significantly increase by 45% and the odds of death before discharge reduce by 30%.^36^

OR - Odds Ratio

There are 15 Academic Health Science Networks (AHSNs) across England, and they operate as the key innovation arm of the NHS. PERIPrem is a product of the merger of two successful, independent West of England AHSN (WEAHSN) ‘Evidence into Practice’ bids, put forward by Professor Karen Luyt (KL; Consultant Neonatologist and Professor of Neonatal Medicine at the University of Bristol) and Dr Sarah Bates (SB; Consultant Neonatologist at Great Western Hospitals NHS Foundation Trust) in 2019. The resultant project encompassed the whole South-West region, recognising the importance of regional collaboration to achieve implementation of the bundle elements.

Recognising bundle complexity, large geography, varied populations and reliance on ownership for success, PERIPrem invested in bringing together perinatal organisations caring for a population of 5 million comprising: 12 NHS Trusts (secondary care hospitals), South West AHSN and WEAHSN, South West Neonatal Operational Delivery Network (SWODN), parent partners, South Western Ambulance Service and clinical experts resulting in the UK’s largest perinatal quality improvement (QI) clinical collaborative.

The Trusts who signed up to PERIPrem were:

- Gloucestershire Hospitals NHS Foundation Trust.
- Great Western Hospitals NHS Foundation Trust.
- North Bristol NHS Trust.
- Northern Devon Healthcare NHS Trust.
- Royal Cornwall Hospitals NHS Trust.
- Royal Devon and Exeter NHS Trust.
- Royal United Hospitals Bath NHS Foundation Trust.
- Taunton and Somerset NHS Foundation Trust.
- Torbay and South Devon NHS Foundation Trust.
- University Hospitals Bristol and Weston NHS Foundation Trust.
- University Hospitals Plymouth NHS Trust.
- Yeovil District Hospital NHS Foundation Trust.

We also have industry partners Hologic & Vygon to thank for their contribution to facilitating PERIPrem.

The PERIPrem project aimed to:

1. Improve rates of care bundle implementation to mother–baby dyads who deliver at <34^+0^ weeks’ gestation using QI methodology.
2. Improve rates of individual element implementation to mother–baby dyads who deliver at <34^+0^ weeks’ gestation using QI methodology.
3. To understand the barriers and enablers associated with the PERIPrem approach on the implementation of a multiple-element standardised bundle.
4. To establish the impact of the PERIPrem approach on perinatal staff knowledge, skills and confidence in QI methodology, psychological safety and teamwork.

Longitudinal aims beyond the scope and time-frame of this current evaluation were to:

1. Reduce mortality and brain injury rates in infants born <34^+0^ weeks’ gestation in the South West of England.
2. Eliminate inequitable access to all elements of the care bundle for socially disadvantaged mothers and babies

## Background

When tasked with providing a core outcome set for research involving premature infants, parents and patients demonstrated their priorities by placing survival, brain injury on imaging, general gross motor ability, general cognitive ability, quality of life, visual impairment/blindness and hearing impairment/deafness in their top 12 outcomes.^7^ A diagnosis of cerebral palsy can have an immeasurable impact on the life of a child and their family with one out of three unable to walk, one out of four unable to talk, one out of four having epilepsy, three out of four experiencing pain and one out of two having an intellectual impairment.^8^ The human importance of a project such as PERIPrem cannot be underestimated. The financial lifetime cost of cerebral palsy has been estimated at £700 000, with an increasing cost to NHS Resolution.^9 10^

Despite conclusive evidence of the effectiveness of many interventions, there remains marked variation in practice throughout the UK when one examines data collected by the National Neonatal Audit Programme via the BadgerNet neonatal clinical care dataset.^11^ Indeed, groups like the British Association of Perinatal Medicine (BAPM) and the Royal College of Paediatrics and Child Health call for QI methodology to be used to reduce variation in practice, which outcomes from projects like PReCePT have proven both feasible and sustainable.^12^

The evidence for each individual element of the PERIPrem programme is contained previously in table 1. It is not possible to quantify the impact of the bundle of interventions as a whole, but each intervention in itself carries significant opportunity to improve the mortality and risk of neurodevelopmental impairment for preterm infants.

PERIPrem aimed to build on the QI theory and the community of PReCePT to optimise rates of compliance to the bundle of evidence-based interventions and to examine whether such methodology could bring perinatal multidisciplinary teams (MDTs) together to aid challenging, complex objectives such as birth in the right place.

There have been multiple QI projects conducted in the past to improve rates of individual perinatal interventions, but to our knowledge, none have been as complex as PERIPrem in its ambition to approach eleven elements in 12 NHS Trusts. Empowering clinicians and parent partners to own the QI journey, supported by regional organisations, increases speed of roll out, maximises capacity and increases access to training. The translation of ideas and ambition into demonstrable improvement was made possible through the collaboration of all organisations offering particular skill sets to the ambition.

## Measurement

The characteristics of the mother–baby dyads who were eligible to enter the PERIPrem project were collected via the PERIPrem Optimisation Tool, a smart Excel spreadsheet. The tool was submitted monthly by each unit to the evaluation team. The tool detailed all eligible babies born that month with information about their demographics to inform bundle eligibility, as well as completeness and timing of bundle elements as detailed in table 1. Teams were encouraged to use the PERIPrem Patient Passport to ease data collection (available here: https://www.weahsn.net/our-work/transforming-services-and-systems/periprem/periprem-quality-improvement -resources/).

In order to understand if adherence to the bundle had changed from preimplementation to postimplementation of PERIPrem two stratified samples were collected. The 12 Trusts were asked to complete a copy of the Optimisation Tool retrospectively for the first ‘*x’* number of babies born after 1 January 2019 (18 months before implementation), a total of 120 babies across all 12 Trusts (special care units=5 babies, local neonatal units=10 babies, NICUs=15 babies). These 120 babies were compared with the last 120 babies (same number of babies from each Trust) born prior to 1 July 2021 (the close of implementation support).

Mixed-effects binary logistic regressions were conducted on this data using a two-level model, where the mother–baby dyad was the primary level of analysis and adjusted for clustering by Trust. Each of the bundle elements, as well as whether the dyad was fully optimised (received all the interventions), were entered into the model as the dependent variable. For the comparison of 2019 and 2021 data, time was added as a fixed factor. For the analysis of the data in the implementation phase, time was added as a continuous factor. For the optimisation score (a continuous variable), linear mixed models were used using the same hierarchical structure. It is important to note that linear mixed models can be limited by multicollinearity of predictors, and care was taken when deciding what predictors were included in the models, and final models were selected using ranking via Akaike’s Information Criterion.^13^

Predictors for each model included: baby characteristics (gestation, birth weight and single or multiple birth) collected at a patient level; maternity characteristics (ethnicity, age and deprivation) collected at a Trust level; and whether or not the unit was a NICU.

In order to understand the human factor mechanisms at play in the implementation of the PERIPrem approach, a range of measures and methods were adopted, including self-reported questionnaires and qualitative semistructured interviews. Quantitative self-reported surveys were administered in September–October 2020 and again in June 2021, via an online survey hosted by SurveyMonkey and included the following questionnaires:

- Psychological Safety using items taken from the Psychological Safety subscale in The Learning Organization Survey.^14^
- Staff Perceptions of Team Work using the TeamSTEPPS Teamwork Perceptions Questionnaire.^15^
- Barriers and Enablers to Implementation using qualitative semistructured interviews with staff and PERIPrem team members alongside an online survey based on the Theoretical Domains Framework for behaviour change, the barriers to QI identified in the published literature and expert opinion.^16^

Scores in the Psychological Safety subscale and the TeamSTEPPS Questionnaire were analysed using linear mixed models (with staff as primary level and Trust as secondary level) and responses to the questions around barriers and enablers to implementing QI methodology analysed using generalised estimating equations.^15^

## Design

Using the QI implementation design of the AHSN team that delivered PReCePT under the leadership of KL and the local, multiple-element care bundle implementation experience of SB, the PERIPrem team assembled.^12^ The project team consisted of perinatal team members (midwives, obstetricians, neonatologists), the AHSN Patient Safety Collaborative team and parental coproduction.

Every Trust in the SWODN was invited to participate in the project. This invitation was extended to each Trust’s executive board, as well as to their perinatal teams. Every Trust accepted the invitation and the PERIPrem team proceeded by:

- Defining the 11 interventions, their evidence base and eligible cohort.
- Understanding the patient population through regional BadgerNet data and parent partner lived experience.
- Attempting to predict barriers to implementation between MDT members, local, regional and national systems.
- Designing resources to comprise data collection, parent and professional information sharing, QI processes and microsystem exploration.
- Supporting local teams with bespoke QI coaching.
- Monitoring progress in real-time, facilitating collaboration.
- Planning the evaluation, dissemination and communication of the findings locally, nationally and internationally.

## Strategy

To structure the project, we employed the established QI Model for Improvement by the Institute for Healthcare Improvement (IHI) and elements of anthropological experience-based codesign.^17 18^

### Design phase

In the spring of 2020, the PERIPrem team set forth with the paragon of parental coproduction to put the voice of patients and families at the heart of the project. Concomitantly, the COVID-19 pandemic gave way to a national lockdown, forcing complete change to the implementation strategy of PERIPrem. The team rallied and adversity gave birth to remote leadership innovation. Employment of the experience-based codesign toolkit led to establishment of an extensive, PERIPrem branded, resource library inclusive of:

### Tools for professionals

- Data collection: the Optimisation Tool to collect standardised monthly patient data.
- PERIPrem bundle real-time intervention checklist: the PERIPrem Patient Passport.
- Infographics: summarising the application and effectiveness (number-needed-to-benefit) of each intervention in the bundle, empowering professionals to make informed cases for change.
- Online video compendium about each intervention and the PERIPrem mission.
- Evaluation tools: the Snapshot tool and Debrief tool to facilitate deeper dives into the processes, barriers and facilitators.
- Drop-in QI sessions with a QI AHSN director.
- Two teams undertook silver QI training during the project using our parent representatives as voices for their own project team.
- The Trusts were also offered an online virtual QI course run from the WEAHSN for free.

### Tools for patients/parents

- A coproduced patient information leaflet on Expressed Breast Milk, translated into the eight most commonly spoken languages in the South-West England.
- A PERIPrem intervention checklist for parents *by parents* so that they are informed of what their baby has received and could expect from the MDT; The PERIPrem Parent Passport was also translated.

### Networking events

- Online Share&Learn sessions consisting of a guest speaker webinar followed by the opportunity to share regional barriers and excellence.
- The PERIPrem online celebration event with promotional materials posted to all units to extend a personal touch when we could not visit in person; this also happened at Christmas.

Operational delivery strategy was also considered; PERIPrem funded £4500 or 90 hours each over the 9-month project implementation phase for two PERIPrem Leads per hospital (a midwife and neonatal nurse). These teams were encouraged to recruit their own local implementation teams in particular with an obstetrician and neonatologist to facilitate change that required certain permissions.

Each region had a funded lead obstetrician and neonatologist, and each Trust was allocated a QI coach with whom they could develop their own relationship based on local-need-specific demand.

An equality impact assessment was performed. The team planned on more extensive community engagement from more vulnerable and marginalised parent groups, but unfortunately, the pandemic hampered this in its execution.

### Implementation phase

**Figure 1:**
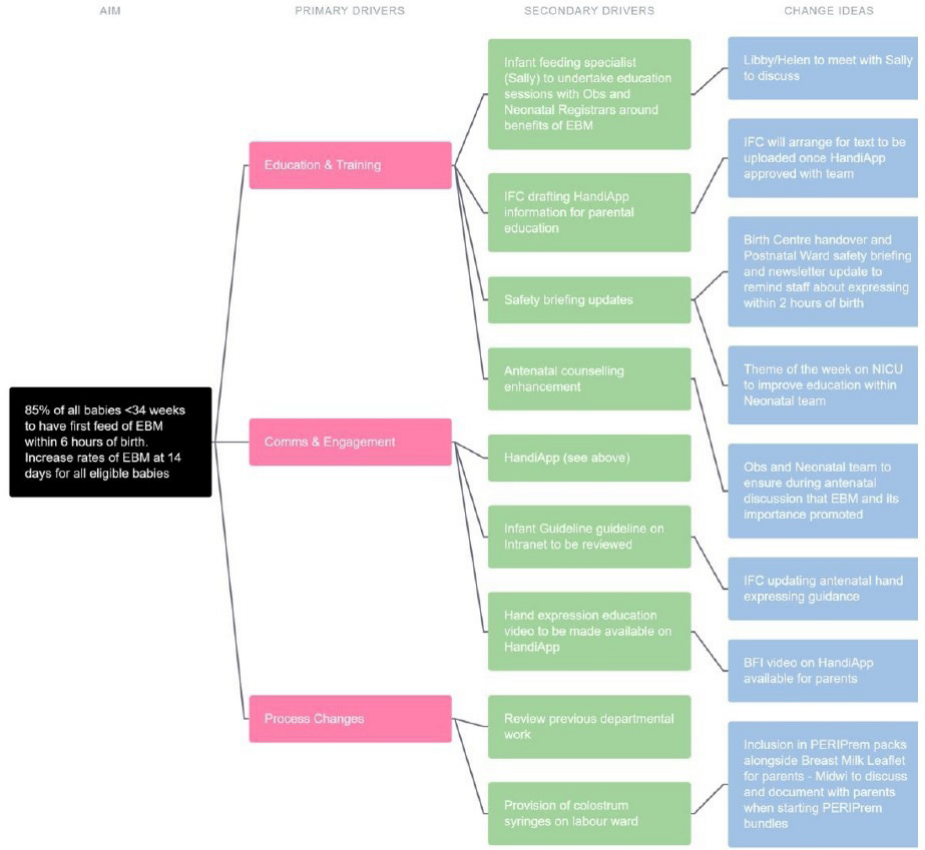
The driver diagram designed on LifeQI by one of the local teams looking at approach to improving early breast milk.

The 12 Trusts joined the project over a staggered 3-month period dependent on local barriers. Team members varied in QI experience and project leadership. Teams met remotely with their QI coaches and the PERIPrem operational lead to establish an action plan and ensure that they were able to access the PERIPrem resources. They tended to choose to focus their efforts on elements where the most local improvement could be made and as this varied, so did the barriers encountered. Plan–Do–Study–Act structured cycles of change were encouraged, but teams were autonomous in their execution in response to their microsystem-specific demands, and in response so was the QI support that they received (figure 1.). The PERIPrem resources were iteratively adapted in response to feedback with a weekly newsletter that was emailed round to teams celebrating regional successes and innovations.

In September 2020, additional funding of £500 per unit was made available via a grant from Hologic. With the help of senior staff PERIPrem leads used this money to bypass complex local procurement routes and create a range of interventions including: PERIPrem grab boxes, resources for tea trolley training, blankets, TransWarmer infant transport mattresses and Neohelp sterile polyethylene suits.

PERIPrem also supported the successful SWODN application to fund quantitative fetal fibronectin testing across all our units for free for a year to support place of birth, encouraging evidence-based QUIPP app calculation of risk of preterm birth, thus hoping to rationalise centralisation of mothers in extremely preterm labour.

Antenatal antibiotic administration to all mothers in established preterm labour was introduced in March 2021 alongside the introduction of optimal timing of the antenatal interventions to the Optimisation tool.

The implementation phase was extended from 6 to 9 months to take into consideration the multitude of barriers that COVID-19 wrought.

### Evaluation, dissemination and spread

The WEAHSN and South-West AHSN commissioned an independent quantitative and qualitative evaluation of the project to examine, in detail, the impact of the PERIPrem approach as a standardised bundle of care as described in the measurement section previously. We also examined its impact on maternity and neonatal staff knowledge, skills and confidence in QI methodology, psychological safety, teamwork and associated barriers and enablers.

Throughout project implementation national and international awareness of PERIPrem was building. The operational (SB) and strategic (KL) leads were invited to speak at a number of events, and free, online availability of our resources hastened the spread of interest prior to availability of much evaluation data. As national programme leads such as BAPM and MatNeoSIP encouraged QI to affect improvement in rates of preterm perinatal interventions, PERIPrem realised the opportunity to collaborate, resulting in PERIPrem becoming the vehicle of improvement supported by MatNeoSIP, ensuring its sustainability.

In the continued spirit of coproduction, a collection of parents whose infants experienced the PERIPrem journey, selflessly consented to recording *their* experience of PERIPrem. The result of these recordings (https://vimeo.com/485976065) was an incredibly emotive chronicle of preterm parent lived experience, which to this day is the best statement exhibiting the motivation for PERIPrem.

## Results

The first aim of PERIPrem was to improve rates of bundle implementation to mother–baby dyads who deliver at <34^+0^ weeks’ gestation using QI methodology. This increased by 26% (from 3% to 29%) between 2019 and 2021. Binary logistic regression found that dyads were significantly more likely to be fully optimised in 2021 compared with 2019 (probability=0.96 (95% CI 0.87 to 0.99), p<0.001). However, there was no significant difference when focusing on the implementation period (probability=0.50 (95% CI 0.48 to 0.52), p*=*0.793).

The second aim of PERIPrem was to improve rates of individual element implementation. The percentage of eligible interventions received by dyads increased by 23% (from 55% to 78%) between 2019 and 2021. The linear mixed model found that the scale of this improvement did not change after adjusting for covariates and was highly statistically significant (p<0.001). However, there was also no significant difference in percentage of interventions received when focusing on the implementation period (p=0.19).

Binary logistic regression found that there were significant improvements for all the individual elements from 2019 to 2021 (ranging from 8% to 63%; p<0.05), apart from magnesium sulfate (+11%; p=0.197), volume-targeted ventilation (+8%; p=0.750) and intrapartum antibiotics (−4%; p=0.351). The lack of change in magnesium sulfate may be due to adherence rates already peaking as a consequence of the PReCePT project, and intrapartum antibiotics was only introduced in March 2021, which meant Trusts had less of an opportunity to bring about change.

**Figure 2:**
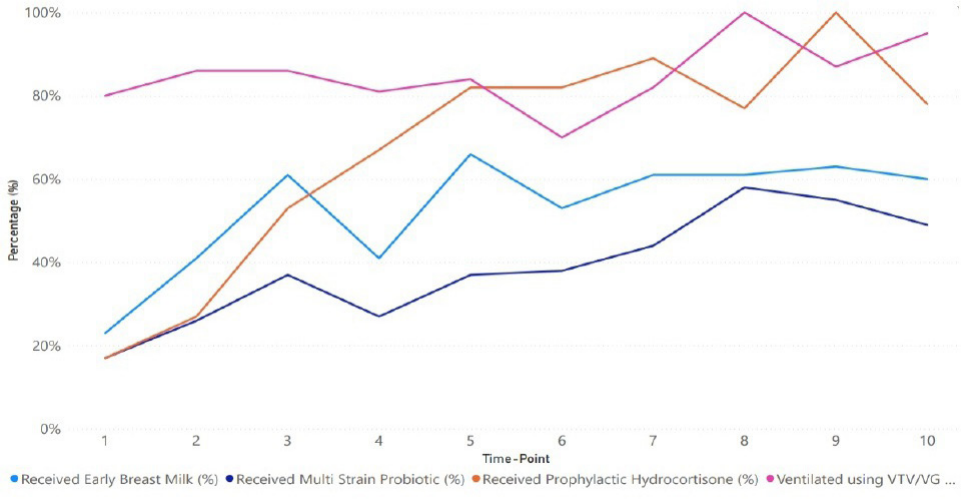
Regional changes over the implementation period in the percentage of babies that received volume-targeted ventilation, breast milk within 6 hours, a multistrain probiotic and prophylactic hydrocortisone.

Binary logistic regression over the implementation period found that there were statistically significant improvements in volume-targeted ventilation (probability=0.53 (95% CI 0.50 to 0.56), p=0.047), receiving early breast milk (probability=0.54 (95% CI 0.51 to 0.56), p=0.003), probiotics (probability=0.57 (95% CI 0.52 to 0.62), p=0.005) and postnatal prophylactic hydrocortisone (probability=0.61 (95% CI 0.55 to 0.66), p<0.001) (figure 2). Although place of birth did show a steady increase, this was not statistically significant (probability=0.61 (95% CI 0.48 to 0.73), p=0.095).

When examining the impact on psychological safety and team-working, linear mixed models indicated a statistically significant improvement in team function (p=0.021), situation monitoring (p=0.029) and communication within teams (p=0.002) over the implementation phase.

## Lessons and limitations

As a change implementation construct, PERIPrem had many strengths both subjectively observed by the qualitative interviews with the local implementation teams and objectively measured through intervention implementation rates. The key strengths recognised were in response to the challenges faced:

COVID-19 placed constraints on staff place and pattern of work, reduced staffing numbers, resulted in significant additional mental load and prevented simulation and in-person collaboration.

Marrying this with the regional expanse of the project, the size of the MDT workforce involved and the scope of the interventions, teams struggled to find the time to implement change *and* report outcomes despite the funded hours, due at least in part to competing demands.

Our strengths:

- Innovation through a blended, remote approach to regional project design and implementation in a time of restricted travel.
- Comprehensive branding and packaging of support resources leading to regional continuity of care and use of a common language.
- Enabling transfer of up-to-date knowledge about the significance of these evidence-based interventions to a whole regional, perinatal workforce through a variety of approaches: infographics, videos and webinars.
- Delivery of real-time, visual data by local teams using the PERIPrem-supplied resources (Optimisation tool & LifeQI), and from the regional evaluation hub to provide teams with contemporaneous feedback on changes implemented.
- Being a realisation of translation of evidence into practice in a short time frame.
- The evidence of ongoing improvement in intervention delivery after the implementation phase and its associated support mechanisms has come to an end suggests successful embedding of change.
- The digital delivery of the project thereby reducing travel and the expected improvement in patient outcomes will result in a decreased carbon footprint.

PERIPrem was a challenging, multiple-element care bundle for Trusts to adopt, but the reality of the project ask was to reduce variation and ensure that every patient gets the best, pre-existing evidence-based care. While we were able to show significant improvements in element delivery between 2019 and 2021, the implementation period itself identified significant improvement in four elements only. The reasons behind this are complex as each intervention process is nuanced with its own barriers and enablers, but one also cannot underestimate the impact that the COVID-19 pandemic had across our region on staff capacity. Without a control group or data on other programmes Trusts were implementing, it is difficult to strictly infer whether the significant improvements observed between the 2019 and 2021 cohorts for several elements of the bundle were as a direct result of PERIPrem, but the contemporaneous relationship of the change is suggestive. National Neonatal Audit Programme data from 2019 to 2020 demonstrates insignificant change nationally in several PERIPrem elements including: birth in the right place for <27 week infants, antenatal steroid administration and normothermia, suggesting against our observed improvements being secondary to comparative practise change over the pandemic.^11^

Maternal characteristics were not available on a patient level, and therefore data obtained at Trust level were used. There were also differences in sample size dependent on the bundle element being analysed due to evidence-based gestation-related eligibility criteria. A further limitation specifically for the analyses of the 2019 and 2021 datasets was that these were stratified samples; however, distribution of the dyad characteristics was similar to the population used in the analysis of the implementation phase.

## Conclusion

The notion of care bundles was first introduced by the IHI, who describe them as containing three to five evidence-informed practices that need to be delivered collectively and consistently to improve patient outcomes.^19 20^ In a scoping review of the barriers and enablers to the implementation of care bundles, a significant association was found between worsening adherence and increasing complexity.^21^ At 11 elements, the complexity and size of the PERIPrem bundle was described by PERIPrem Leads as a barrier to implementation and the narrative in the interviews very much indicated that the bundle was seen as 11 individual practices. There are examples of complex neonatal care bundles that have been successfully implemented and achieved improvement neurological outcomes for preterm babies.^22^ The evaluation of the coproduction and implementation of a complex care bundle spanning antenatal, intrapartum and postnatal care, delivered by multispecialty teams in a regional perinatal network, is novel.

We have been able to demonstrate significant improvements in adherence to the PERIPrem bundle and its elements from 2019 to 2021 and also within the implementation phase of the project. The project achieved this through implementation innovation, improvements in regional perinatal team culture, refreshing staff evidence-based knowledge and skills and access to comprehensive support and resources. The evaluation has provided evidence-based knowledge and recommendations that have advanced our understanding of the processes that underpin the successful implementation of PERIPrem that we will take forward in its spread as a vehicle of MatNeoSIP.

The sustainability of PERIPrem will manifest through its role in the facilitation of the MatNeoSIP programme in the South West and the routine reporting of element implementation to the SWODN. Further analysis of these results after a year will provide us with more long-term data. PERIPrem has strongly supported project dissemination through open access release of all resources online (https://www.weahsn.net/our-work/transforming-services-and-systems/periprem/), and National dissemination feasibility using the AHSN network has previously been modelled by PReCePT.^23^

We await the long-term mortality and severe brain injury impact data of PERIPrem, but the evidence behind these interventions suggests that PERIPrem is an ideal vehicle with which to achieve UK-wide 50% reduction in neonatal mortality in brain injury by 2025.

## Patient and public involvement

### How was the development of the project and outcome measures informed by patients’ priorities, experience and preferences?

Parent partners were involved from the outset of the design of the project. They were invited to participate at every stage and came up with key project outputs such as the PERIPrem Parent Passport, running their own Share&Learn webinar and through their networks putting the project team in contact with other parents for parent voice resources. They have been truly integral.

### How will the results be disseminated to project participants?

The results of PERIPrem will be disseminated after publication via the AHSNs, the PERIPrem Twitter account, our parental coproducers and their respective charities (SNUG) and to our 12 participating units in full report and summary infographic form.

## Data Availability

No data are available. Not Applicable.
